# A new method to measure complexity in binary or weighted networks and applications to functional connectivity in the human brain

**DOI:** 10.1186/s12859-016-0933-9

**Published:** 2016-02-13

**Authors:** Klaus Hahn, Peter R. Massopust, Sergei Prigarin

**Affiliations:** Institute of Computational Biology, HMGU–German Research Center for Environmental Health, Ingolstädter Landstraße 1, Neuherberg, 85764 Germany; Centre of Mathematics, Research Unit M6, Technische Universität München, Boltzmannstrasse 3, Garching bei München, 85747 Germany; Novosibirsk State University, Institute of Computational Mathematics and Mathematical Geophysics, Siberian Branch of Russian Academy of Sciences, Novosibirsk, Russia

**Keywords:** Network analysis, Complexity measure, Computational algorithm, Fractal dimension, Human brain application, Functional connectivity

## Abstract

**Background:**

Networks or graphs play an important role in the biological sciences. Protein interaction networks and metabolic networks support the understanding of basic cellular mechanisms. In the human brain, networks of functional or structural connectivity model the information-flow between cortex regions. In this context, measures of network properties are needed. We propose a new measure, Ndim, estimating the complexity of arbitrary networks. This measure is based on a fractal dimension, which is similar to recently introduced box-covering dimensions. However, box-covering dimensions are only applicable to fractal networks. The construction of these network-dimensions relies on concepts proposed to measure fractality or complexity of irregular sets in $\mathbb {R}^{n}$.

**Results:**

The network measure Ndim grows with the proliferation of increasing network connectivity and is essentially determined by the cardinality of a maximum *k*-clique, where *k* is the characteristic path length of the network. Numerical applications to lattice-graphs and to fractal and non-fractal graph models, together with formal proofs show, that Ndim estimates a dimension of complexity for arbitrary graphs. Box-covering dimensions for fractal graphs rely on a linear log−log plot of minimum numbers of covering subgraph boxes versus the box sizes. We demonstrate the affinity between Ndim and the fractal box-covering dimensions but also that Ndim extends the concept of a fractal dimension to networks with non-linear log−log plots. Comparisons of Ndim with topological measures of complexity (cost and efficiency) show that Ndim has larger informative power. Three different methods to apply Ndim to weighted networks are finally presented and exemplified by comparisons of functional brain connectivity of healthy and depressed subjects.

**Conclusion:**

We introduce a new measure of complexity for networks. We show that Ndim has the properties of a dimension and overcomes several limitations of presently used topological and fractal complexity-measures. It allows the comparison of the complexity of networks of different type, e.g., between fractal graphs characterized by hub repulsion and small world graphs with strong hub attraction. The large informative power and a convenient computational CPU-time for moderately sized networks may make Ndim a valuable tool for the analysis of biological networks.

## Background

Network or graph theory is of increasing importance for the analysis of biological systems. This may comprise protein interaction networks [1], metabolic networks [2] or neuronal networks in the human brain. Task-induced and resting state functional magnetic resonance imaging (fMRI) and diffusion tensor imaging (DTI) established the analysis of functional and structural connectivity networks in the human brain [3, 4]. For quantitative analyses, local and global topological measures are employed; see [3, 5, 6] for a description and a critical discussion. We introduce in this paper a new concept, which may be called a “regional quantity” and which measures the complexity of networks. The problem how to measure complexity of a graph has been approached in different ways. For example, complexity of a graph has been defined by the number of its spanning trees [7]. It has been defined as the number of Boolean operations to construct the graph from generating graphs [8]; this type of complexity is frequently called computational complexity. Or, it has been defined by a combination of the number of vertices, edges and proper paths [9]. Our concept of complexity is based on the connectivity of a graph. Simple examples of this type of complexity are the cost or the efficiency, more involved examples are the box-counting dimensions [10, 11] introduced recently. Cost and efficiency are easily calculated, however both concepts fail to discriminate regular grids with different dimensions, see Ndim and manifolds. Box counting-dimensions can detect a novel type of graphs, the fractal graph; however, these dimensions need NP-complete algorithms and become unstable if fractality is distorted [12]. In our approach we try to overcome these shortcomings by a new concept, which is applicable to any undirected graph. We achieve with our concept more flexibility and enhanced informative power, the price however is again a NP-complete algorithm with high CPU times for some large sized networks. Practical applicability of Ndim to such cases will be demonstrated by the introduction of convenient lower bounds.

Our concept is based on a definition of a fractal dimension introduced for sets in $\mathbb {R}^{n}$ by Sandau [13] and Sandau & Kurz [14]. Fractal analysis in $\mathbb {R}^{n}$ allows the quantification of irregularity or complexity of point sets where the concepts of traditional geometry usually fail. Fractals possess a fine structure that exhibits details at different scales of resolution. Fractals appear in numerous disciplines, e.g., plasma physics, biological systems, or neuroscience [15–17].

The degree of complexity is measured by a fractal dimension *FD* although many non-equivalent definitions of *FD*s exist. However, all such concepts of *FD* satisfy (at least approximately) the following unifying conditions, see [13, 15].

1. Invariance under Euclidean motions;
2. Invariance under affine transformations;
3. Monotonicity for set inclusion;
4. Maximum property for set union;
5. *FD*s are extensions of the topological dimension for smooth manifolds, and
6. All *FD*s are equivalent for self-similar fractals.

A picturesque definition of self-similarity is due to Mandelbrot [18]: A fractal is called *self-similar* if a subset, magnified to the size of the whole set, is congruent to the whole. (See [15] for a formal definition and extensions.) The numerical calculation of *FD*s for real data with scale of resolution >0 is, in general, nontrivial as the details of scales tending to zero are important for the characterization of a fractal [17]. In [13, 14] an *FD* definition, called x-dim, was proposed that is both applicable to self-similar and more general fractals. This definition appears to be numerically more robust than the frequently used box-counting dimension for self-similar fractals.

Using a procedure similar to the calculation of a box-counting dimension in $\mathbb {R}^{n}$, a novel *FD* for networks or graphs was introduced by Song et al. [10, 11]. For this purpose, a covering of the graph by subgraph (“box”) systems differing in their linear size is proposed. The *FD* is finally determined by the slope of a linear log− log plot, where the minimal number of covering subgraphs is plotted versus their linear size.

Networks have in general no well defined geometric patterns that allow the definition of self-similarity. But self-similarity can be defined by internal properties of the graph, e.g., by the invariance of the degree distribution under scale transformations. To obtain graphs on different length scales, a subgraph covering with fixed linear size is iterated, blurring the initial graph more and more, thus increasing the scale; see Song et al. [10, 11]. If the degree distribution is invariant under the different steps of this renormalization procedure, the graph is called self-similar [19, 20]. A similar scaling invariance for edge densities was discussed by Blagues et al. [21]. Note the following difference to fractals in $\mathbb {R}^{n}$: There exist self-similar graphs which are not fractals, i.e., graphs for which the log− log plot is nonlinear [11, 20].

Inverting this iterated renormalization procedure allows the design of models for fractal and non-fractal scale free graphs [11, 12]. Transitions between both classes depend on the strength of the module or hub repulsion which is controlled by the model parameters; minimal hub repulsion (attraction) produces a model with small world properties, maximal hub repulsion a fractal graph [11]. It is shown in [12], that the addition of noise by adding random edges can also initiate the transition from a fractal to a small world graph. Many real data networks are fractal graphs; examples are the world-wide-web (WWW), social networks, protein-protein interaction graphs (PIN), and cellular networks [10, 11]. More recently, Gallos et al. [22, 23] detected fractal fMRI networks in the human brain for high percolation thresholds. An alternative to the box-covering methods is discussed in [24]; they use a random walker through the network, to derive a correlation dimension using a convenient log− log plot.

In this paper, we propose an extension of Sandau‘s fractal dimension x-dim in $\mathbb {R}^{n}$ to networks or graphs, which we call Ndim. This new concept is based essentially on the maximum *k*-clique cardinality and does not only allow the quantification of complexity for fractal graphs, but also for graphs with non-linear log− log plots. We prove that this concept satisfies the graph-specific modifications of the conditions C2, C3, and C4; condition C1 is irrelevant for graphs. The validity of conditions C5 and C6 is demonstrated numerically by applications of Ndim to regular lattice or grid graphs and to binary fractal models. As weighted graphs are frequently used to quantify functional or structural connectivity, we present three procedures to apply Ndim to weighted graphs. To evaluate the complexity Ndim, we compare it with the connectivity measures cost, efficiency, and box-counting dimensions by applications to resting state and task-induced fMRI data. We find several advantages of our new measure: compared to cost and efficiency Ndim has stronger informative power, as complexity differences in fMRI correlation networks between healthy and depressed subjects are increased for Ndim. In contrast to cost and efficiency is Ndim a non-global regional metrics. We show that this feature enables the localization of special hub nodes in the networks which are characteristic for depressed subjects. Comparing Ndim and some box-counting metrics, we find a strong similarity as long as the networks are fractals; applying these concepts to experimental dual task fMRI networks, which lose fractality by lowering a correlation threshold, we find that Ndim has an essentially enhanced scope of application compared to box-counting dimensions. Though the algorithm to calculate Ndim is NP-complete, we find that CPU time is quite low for moderate network sizes, for large sized networks we discuss the introduction of lower bound constraints.

## Methods

### Extended counting method in Euclidean space

**Fig. 1:**
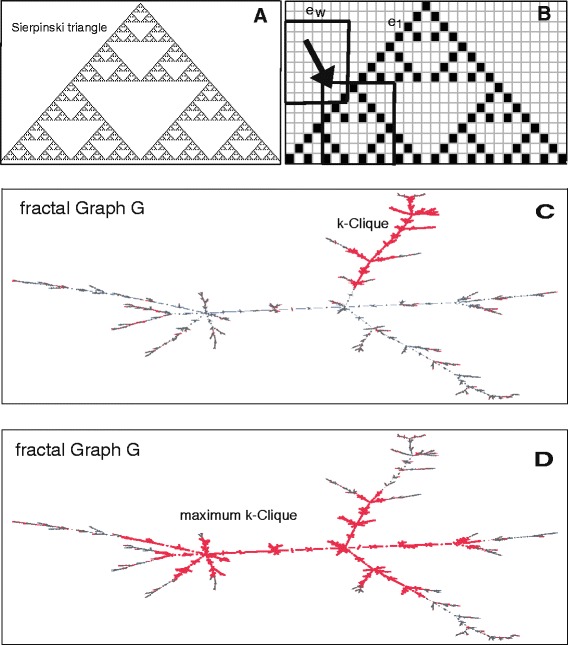
Panel **a** Sierpiński triangle *F* in the plane. Panel **b** Extended counting method for *F*. The scales *e*_1_ and *e*_*w*_ for the fine grid and the sliding window (*thick frames*) are indicated. The window with the maximum number of fine grid boxes intersecting *F* determines the complexity of the fractal (x-dim). Panels **c** and **d** show a fractal graph *G* with a submaximum (**c**) and a maximum (**d**) highlighted *k*-clique (*thick red*). The cardinality of a maximum *k*-clique determines the complexity of *G* (Ndim(*G*))

For a point set in $\mathbb {R}^{n}$, the fractal dimension x-dim is numerically calculated using the extended counting method [13]; see Fig. 1a, b for an illustration.

In Fig. 1a, b, the point set *F* (Sierpiński triangle) is covered by a fine uniform grid with grid scale *e*_1_ satisfying *e*_1_≥ the resolution of the data points given in Fig. 1a. An additional large window with size *e*_*w*_= (minimal side length of the wrapping box of *F*)/2 is slid along the corner points of the fine grid to find the maximum intersection between *F* and the fine grid within the window; see Fig. 1b. Denoting the maximum number of intersecting fine boxes by *N*, x-dim is then estimated by

$${\textrm{x-dim}} (F) = \frac{\log N}{\log (e_{w}/e_{1})}. $$

(See [13, 17]). For x-dim estimates of MRI cortex surfaces, fMRI time series in the human brain, and fractional Brownian motion, we refer to [25–27].

### Extended counting method for networks

For a connected binary network or graph *G*, we define an extended counting method similar to that for a point set in $\mathbb {R}^{n}$. The fractal dimension Ndim of *G* is computed in several steps:

1. Compute the average distance *μ* of *G*. The average (chemical) distance, characteristic path length, or normalized Wiener index is a natural measure for the compactness of a graph [28, 29]. It is defined by
  $$\mu := \frac{{\sum\limits_{1\leq i < j \leq n}} d(i,j)}{\binom{n}{2}}, $$
  where *n* denotes the number of vertices and *d*(*i,j*) the chemical distance or shortest path between vertices *i* and *j*.
2. Reduce *μ* to an integer *k*:=⌊*μ*⌋. Here ⌊·⌋ denotes the floor function.
3. Compute a vertex set that is defined by a maximum *k*-clique of *G*. A maximum *k*-clique is a largest set of vertices with distance ≤*k* in *G* to each other. Such a clique can be interpreted as a cluster, module, or cohesive subgroup of vertices [30].
4. We define Ndim for a finite graph *G* by
  $${\text{Ndim}} (G) = \frac{\log |\max {\textrm{\textit{k}-clique}}|}{\log(k+1)}, $$
  where | max*k*-clique| denotes the cardinality of a maximum *k*-clique. Following the convention of Song et al. [10], we added a +1 to the chemical distance in the denominator.
5. For infinite graphs *G* with arbitrary large *k*, a fractal dimension Ndim can be defined by
  (1) $$\begin{array}{*{20}l} {\text{Ndim}} (G) = {\lim}_{\textit{k}\to\infty} \frac{\log |\max {\textit{k}-\textrm{clique}}|}{\log(k+1)},\\ \quad \text{provided this limit exists.} \end{array} $$
  The above definition is similar to the definition of fractal dimensions for sets in $\mathbb {R}^{n}$ where an infinitesimal process is applied [15]; see Ndim and manifolds and Applications of Ndim to fractal and non-fractal models for examples of graphs where the number of vertices and *k* go to *∞*.
  See Fig. 1c, d for an illustration of a sub-maximum and a maximum *k*-clique in a fractal graph. As will be shown in later sections, Ndim is a fractal dimension *FD* in the sense that it satisfies the conditions C2 – C6 for graphs given in. For simplicity, the notion of fractal dimension is also used for Ndim when applied to finite graphs.

The numerically most difficult part in the computation of Ndim(*G*) is the calculation of the maximum *k*-clique where a scan through *G* is necessary. This is a NP-complete problem [30] just as the computation of box-coverings for the *FD* of [10]. For the clique computations, an algorithm by Carraghan & Pardalos [31] and an algorithm by Tomita et al. [32] applied in the commercial software package Mathematica are used. For a comparison, the box-covering dimensions defined via log− log plots are also computed. We employ the maximum-excluded-mass-burning (MEMB) algorithm and a compact-box-burning (bcm) algorithm as described in [33]. The MEMB algorithm is published online and can be found at http://www-levich.engr.ccny.cuny.edu/webpage/hmakse/brain/.

### Application of Ndim to weighted graphs

The *FD* Ndim was introduced for a binary graph. However, numerous problems in biology or brain research deal with weighted graphs [34]. (Note that all graphs considered in this article are undirected and have no self loops).

In the following, we describe three procedures of how to apply Ndim to weighted graphs with nonnegative weights. Elaborate examples for human brain data and the conditions for applicability are discussed in Results. To obtain a reasonable distance measure *d*(*i,j*) for weighted graphs, the interpretation of the weights *w*(*i,j*) in the weighted adjacency matrix must be considered. For better readability, we do not delineate in the following between a graph and its adjacency matrix.

For “transportation networks,” distances increase with the weights; for “communication networks,” distances decrease with increasing weights [35, 36]. For brain mapping, “communication networks” are frequently used. Here the weights may be given by *w*(*i,j*)=|correlation(*i,j*)| for fMRI time series in two different ROIs *i* and *j* [37], or by *w*(*i,j*)=connectivity-strength(*i,j*) for DTI fiber connection between functional ROIs *i* and *j* [38]. In these cases Ndim can be computed as follows.

1. Transition to a binary adjacency matrix using a cut-off: A mapping from the weighted adjacency matrix *W*_*n*×*n*_ of a graph with *n* vertices to a binary adjacency matrix *A*_*n*×*n*_ with components *a*(*i,j*) is defined by thresholding of some quantity *τ*>0, as follows. If for an edge *w*(*i,j*)>*τ*, then *w*(*i,j*) is replaced by *a*(*i,j*)=1; otherwise *w*(*i,j*) is replaced by *a*(*i,j*)=0. This implies that for, e.g., |correlation(*i,j*)|>*τ*, *d*(*i,j*)=1, and for |correlations(*i,j*)|<*τ*, *d*(*i,j*)>1, as the connecting paths are no longer direct links.
  If the binary graph *A* is disconnected then Ndim is computed for every connected binary component $A_{k_{l}}$, where *k*_*l*_ is the average distance in component *A*_*l*_. The list of *FD*s (Ndim_1_, Ndim_2_…) is combined into a weighted average
  $${\text{Ndim}} (W,\tau) = \sum_{l} \text{weight}(l) \,\cdot\,{\text{Ndim}} (A_{k_{l}}), $$
  describing the complexity of the disconnected graph. As weights, we choose weight (*l*)=(number of nodes in component *A*_*l*_)/*n*, where *n* denotes the size of the entire graph, which reduces the weight for smaller components as they carry less information. Components with only a few nodes should be excluded from the averaging process but the normalization $\sum _{l} \text {weight}(l) = 1$ should be maintained.
2. A Monte Carlo ensemble method: The weighted adjacency matrix *W*_*n*×*n*_ is normalized by setting $\widetilde {w}(i,j) := w(i,j)/\max \{w(i,j) : i,j = 1, \ldots, n\}$ and these weights are interpreted as probabilities. A uniform random number generator assigns to each edge a random number *p*∈(0,1] and defines a mapping to a binary random matrix via the following condition: If $p\leq \widetilde {w}(i,j)$ then *a*(*i,j*)=1; else *a*(*i,j*)=0. By this procedure, an ensemble of binary random graphs is produced, where for large $\widetilde {w}(i,j)$ short distances are frequently randomly generated and for small $\widetilde {w}(i,j)$ mainly large distances [39, 40].
  For every such binary random graph the *FD* Ndim can be computed. In case a random graph is disconnected, a weighted averaging as in (1) can be applied.
3. Calculation of Ndim via functional distances: To calculate statistical measures of weighted connected communication networks *W*_*n*×*n*_, a functional (physical) distance network $\widetilde {W}_{n\times n}$ is often introduced in order to avoid long pathways for strong connections [35, 36, 41, 42]. In the following we adapt this approach for the computation of the complexity Ndim.
  - The *w*(*i,j*) coefficients (edges of a weighted graph) are mapped to functional distance coefficients via, e.g., $\widetilde {w}(i,j) = 1/w(i,j)$.
  - The graph $\widetilde {W}_{n\times n}$ is mapped to an approximating multigraph *M*_*n*×*n*_ with integer weights by scaling by a large factor *c* and rounding [35]: $M_{n\times n} = \lfloor c \widetilde {W}_{n\times n}\rfloor $. Finally, the coefficients *m*(*i,j*)=*∞* are mapped to *m*(*i,j*)=0; see Results for a numerical example of scaling. Then compute, for instance by the Dijkstra algorithm [43], the distance matrix *D*_*n*×*n*_ with coefficients *d*(*i,j*) for *M*_*n*×*n*_, the average distance *μ*, and the minimum positive distance *m*.
  - Transform the multigraph *M*_*n*×*n*_ to a binary distance graph *G*_*n*×*n*_ [44] using the following condition: If *d*(*i,j*)≤⌊*μ*⌋ set *g*(*i,j*)=1; otherwise set *g*(*i,j*)=0 (*g*(*i,i*)=0). For this binary graph calculate a maximum clique if *G*_*n*×*n*_ is connected; otherwise compute a maximum clique in each connected component and use from these maximum cliques the one with maximal vertex cardinality; see an example in the comment for Fig. 8.
  - The complexity Ndim for *W*_*n*×*n*_ is finally estimated by
    $${\text{Ndim}} (W) = \frac{\log |\textrm{maximum clique}|}{\log [(\lfloor\mu\rfloor + m)/m]}. $$
    In case *M*_*n*×*n*_ is a binary graph, Ndim agrees with the definition given in Extended counting method for networks.

### Data

An application of thresholding and of the Monte Carlo ensemble method to compare the complexity of weighted networks relies on data from [45]: For patients with recurrent depression and for healthy controls, a whole brain functional connectivity network was derived from preprocessed resting-state functional MRI data. By anatomical parcellation of the whole brain using the Harvard-Oxford atlas (FSL, Oxford University) 112 regions of interest (ROIs) or network nodes were defined. Time series of functional MRI signals were extracted from each voxel and subsequently averaged within each of the 112 ROIs. A maximal overlap discrete wavelet transform was applied to decompose the regional time series into different frequency scales [46]. Absolute wavelet correlation coefficients at the low-frequency scale (0.060–0.125 Hz) were used to obtain a 112×112 weighted connectivity matrix representing an individual whole-brain functional correlation network for each subject; a similar procedure is used in [37].

A second data set from [22, 23] uses visual and auditory stimuli with four different onset delays to achieve whole-brain dual-task fMRI time series. The phase of the BOLD signal was computed for each voxel *i* and for 40 trials [47]. The phase-based correlations between different voxels *i* and *j* were averaged over the trials. This resulted in a whole-brain correlation matrix representing a weighted subject-specific functional network. This whole-brain network was reduced by a mask of ∼ 60.000 voxels, where only voxels with high activation probability were kept. The data are published online at http://www-levich.engr.ccny.cuny.edu/webpage/hmakse/brain/. The variation of a correlation threshold produces a percolation process: highly correlated voxels indicate separated modules with strong functional internal links, for lower thresholds the modules are merged and the network approaches a small-world topology.

Seven binary connectivity networks, taken from http://www.brain-connectivity-toolbox.net/, are tested for computational cost. Autobahn has a connection 1, if two locations are directly connected by the highway [48]. Air500 summarizes the flight connections between 500 air ports [49]. Three biological networks are: Celegans describing neuronal connections [50], Mac95 and Macaque summarizing corticocortical connections [51].

## Results

In the next section, we numerically explore how well Ndim and a box-covering dimension approximate the dimensions of grid graphs (condition C5). Then, the equivalence between Ndim and several box-covering dimensions is explored for models of self-similar fractal graphs (condition C6). An examination of non-fractal graphs demonstrates that Ndim is applicable beyond fractality, i.e., to graphs with non-linear log− log plots for minimum numbers of covering boxes versus box size. Three methods of how to apply Ndim to weighted graphs on the basis of analyses of functional connectivity in the human brain are explained. Resting state fMRI data of healthy and depressed subjects and dual-task fMRI data are involved to evaluate Ndim.

### Ndim and manifolds

To demonstrate, that the proposed *FD* Ndim is an extension of the topological dimension for smooth manifolds (condition C5 in the introduction), we applied Ndim to regular one- and two-dimensional finite lattice graphs of size 100, 1500, 30×30, 40×40, 80×80, and 100×100. For comparison, a box-covering dimension was calculated applying the method MEMB [33] and the measures cost (global normalized edge density) and global efficiency, which are in use as simple measures of complexity. We refer to Table 1 for the results, which show a convergence of the *FD*s to the topological dimensions as size increases. For cost and efficency we find that they tend to zero for both grids as their sizes increase.

**Table 1:** Ndim compared with MEMB, cost and global efficiency for some one- and two-dimensional grids

	100	1500	30×30	40×40	80×80	100×100
Ndim	0.99	1	1.77	1.79	1.83	1.84
MEMB	0.99	1	1.63	1.69	1.77	1.81
Cost	0.02	0.0013	0.0043	0.0024	0.0006	0.0004
Efficiency	0.085	0.0092	0.077	0.058	0.03	0.023

The log− log plots for MEMB showed a high linearity in all cases. The coefficient of determination *R*^2^ was found to satisfy *R*^2^>0.99. The box sizes for the log− log plots were averaged, as proposed by Song et al. [33], thus increasing the *R*^2^ values. To keep computation time within limits, we did not use adjacency matrices with more than 10,000 nodes. These results show the enhanced informative power of the fractal dimensions as compared to the topological measures.

### Application of Ndim to fractal and non-fractal models

**Fig. 2:**
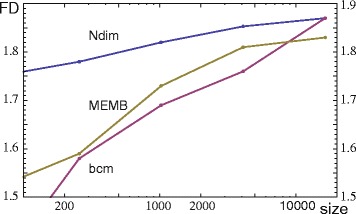
The fractal dimensions Ndim (*blue*), MEMB (*yellow*), and bcm (*red*) are plotted against the size of the network for stages *g*=4,5,6,7 (points from *left* to *right*). The applied model is *f*(*g*,4,2,0). With increasing *g*, all fractal dimensions approach *FD* = 2

A model for scale-free fractal networks was introduced by [11]; it is shown in the Supplement of this paper that this model is in addition self-similar. An explicit algorithm, calculating the model by inversion of a renormalization process, can be found in [11, 12]. In this algorithm, the network grows iteratively in size (number of nodes) whereby the diameter and the degree per node approach the full model. To explore if Ndim satisfies condition C6 for binary graphs, the network dimensions Ndim, MEMB, and bcm are compared at different stages of a growing network. Starting the network construction with a single node, these dimensions are plotted against the size of the model *f*(*g,n*,*m,e*) for *g*=4,5,6, and 7 iterations; see Fig. 2. The growth factor of the network size is *n*=4 per iteration, the growth factor of the degree is *m*=2, the probability for hub attraction is *e*=0; see [11] and [12] for a detailed discussion of the parameters. The *FD* of a full model (*g*→*∞*) can be calculated by *FD*= ln(*n*)/ ln(*m*).

Figure 2 indicates that all numerically computed dimensions approach the value *FD* = 2 with increasing *g*. For the complexity Ndim, an analogous convergence behaviour was observed for the fractal models *f*(*g*,6,2,0), *f*(*g*,5,2,0), *f*(*g*,3,2,0), *f*(*g*,5,3,0), and *f*(*g*,6,3,0). To keep computer time within limits the size of the graphs was restricted to 4^7^ nodes.

**Fig. 3:**
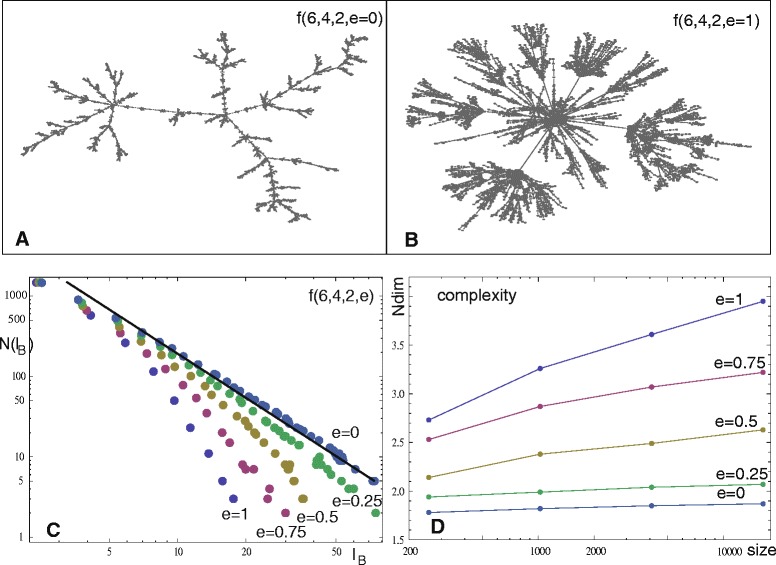
In panel **a**, a fractal and in panel **b** a non-fractal network are presented. Panel **c** shows the number *N*(*l*_*B*_) of covering boxes versus their mean linear size *l*_*B*_ for the models *f*(6,4,2,*e*). Linear regression for *e*=0 is indicated by a straight line. Panel **d** shows the complexities Ndim for *f*(*g*,4,2,*e*) with *g*=4,5,6,7 (points from *left* to *right*), and the indicated values of *e*

Applying the hub attraction parameter 0<*e*≤1, we can construct non-fractal models with nonlinear log− log plots for box-covering dimensions; increasing *e* converts the network more and more to a small world network [19]. Although box-covering dimensions are not well defined for the entire range of log− log plots, Ndim can still be applied in these cases to quantify the complexity of such graphs. The notion of complexity is introduced as an extension of the notion of fractality; see Discussion for an in-depth explanation. Depending on the size, the complexity of a binary network is limited by the complexity of the corresponding complete graph, where Ndim= log(size)/ log2. See Fig. 3a, b for the models *f*(6,4,2,0) and *f*(6,4,2,1).

In Fig. 3c, we show log− log plots for the minimum number of covering boxes *N*(*l*_*B*_) against the averaged box sizes *l*_*B*_, calculated by the MEMB algorithm for models with *e*=0,0.25,0.5,0.75, and 1. For *e*>0, deviations from linearity are clearly visible. The computed complexities Ndim of these models are presented in Fig. 3d for the iterations *g*=4,5,6,7. It is shown in [12], that the addition of small fractions of random edges to the fractal models (*e*=0) produces similar deviations from linearity in the log− log plots.

### Application of Ndim to weighted graphs by thresholding

**Fig. 4:**
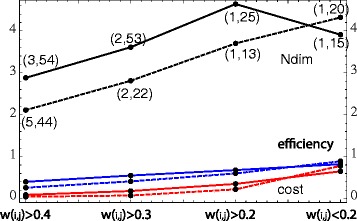
Cost (*red*), efficiency (*blue*) and Ndim (*black*) for a healthy (*broken lines*) and a depressed (*solid lines*) subject are presented for different cut offs indicated. For Ndim, in brackets (*k* = Floor[characteristic path length], cardinality[maximum *k*-Clique]) are given

**Fig. 5:**
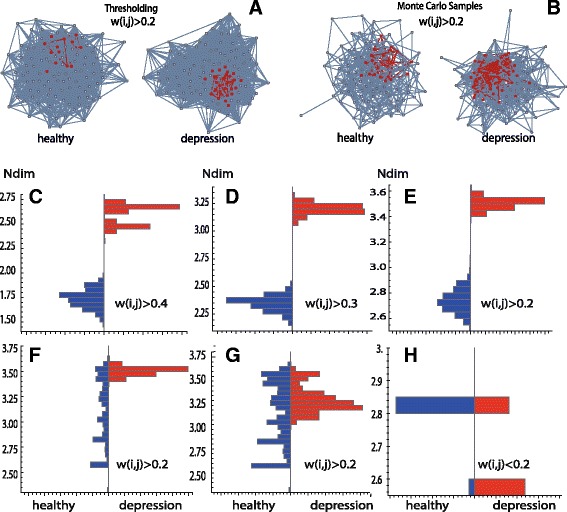
Panel **a** depicts the binary graphs for a healthy and a depression subject computed by the thresholding method. Panel **b** shows binary samples of the Monte Carlo method. The *k*-cliques are indicated in red. Panels **c**, **d**, **e**, **h** represent paired Monte Carlo distributions of complexity Ndim for a healthy (*blue*) and a depression subject (*red*). Panel **f** represents the distributions for a healthy group (*blue*) and the depression subject (*red*), Panel **g** for two groups. The thresholding for the weighted networks is indicated

Abnormal functional brain connectivity is reported for recurrent depression patients compared to the connectivity of healthy subjects [45, 52]. We use such connectivity data to exemplify the informative power of Ndim and to demonstrate how to apply this measure for weighted networks; a detailed clinical study is beyond the scope of this paper. In this section, for a healthy and a strongly affected recurrent depression subject after 9 episodes of depression and a high clinical Hamilton Rating scale HAM-D=23 [45], resting-state fMRI correlation networks of size = 112 as described in Data, are compared. To calculate Ndim and the frequently used topological complexity measures cost (global edge density) and normalized global efficiency, the two weighted absolute correlation networks are transformed to binary graphs by thresholding the correlations *w*(*i,j*), following the procedure outlined in Methods. This procedure is applied for *τ*=0.2,0.3, and 0.4. For *τ*=0.2, the low correlation edges are also filtered out using the following inverse condition: If *w*(*i,j*)<*τ*, then *w*(*i,j*) is replaced by *a*(*i,j*)=1; otherwise by *a*(*i,j*)=0 (*a*(*i,i*)=0). The results are shown in Fig. 4, broken lines belong to the healthy subject, solid lines to the subject with depression. Cost, efficiency and Ndim increase for *w*(*i,j*)>*τ*, with decreasing tau; all measures Ndim are increased for the depressed subject as compared to the healthy subject. Vice versa, for the low correlation graphs *w*(*i,j*)<0.2, the network of the healthy subject is more complex. In general, we find for Ndim, that the differences are more pronounced than for the two global measures, indicating larger informative power of Ndim. For the depressed subject, Ndim decreases from the case *w*(*i,j*)>0.2 to the case *w*(*i,j*)<0.2, whereas cost and efficiency are increasing. This dependency excludes a simple correlation or redundancy between Ndim and the topological measures. See Fig. 5a for binary networks derived by the condition : *w*(*i,j*)>0.2. The left-hand side shows the graph of the healthy subject, the right-hand side the graph of the subject with depression, maximum k-cliques are indicated in red.

### Monte Carlo ensemble method

The same two data sets used in the last section and extensions to groups of 10 healthy controls and 6 depressed subjects (more than 8 episodes and HAM-D> 15) are now analyzed for Ndim by the Monte Carlo ensemble method. To provide a closer comparison with the results of the thresholding method, the weighted networks are also thresholded for a given *τ* before transforming them to the binary random graphs, as is described in Methods. Thresholding is performed like follows, in case *w*(*i,j*)>*τ*, *w*(*i,j*) remains unchanged, otherwise *w*(*i,j*)=0; or, for the low correlation case, if *w*(*i,j*)<*τ*, *w*(*i,j*) remains unchanged, and set otherwise *w*(*i,j*)=0. For a quantitative comparison between the binary ensembles the weighted networks are normalized to probabilities by the total maximum weight collected over all networks involved in the comparison. In Fig. 5b, samples of the binary Monte Carlo ensembles after identical thresholding are presented. The graphs in panel b increase in complexity from healthy to disease as in the cases shown in Fig. 5a. A reduction of connectivity in the Monte Carlo samples compared to panel a, is due to the fact that this method transforms lower *w*(*i,j*) less frequently into the edges of the binary networks. For every weighted network an ensemble of 1,000 binary random networks is calculated and Ndim of a weighted graph is thus quantified by a distribution of complexities; see Fig. 5c–h for examples. The blue distributions belong to the healthy subject, the red ones to the subject with depression. The afore-mentioned thresholding of the weighted graph is indicated in the Figure. Panels c), d), e), h) present comparisons of the two subjects, panel f) a comparison between the control group and the depressed subject, and panel g) the group-group comparison. Statistical subject-subject comparisons may be helpful in personalized medical analysis. The group distributions are involved to reduce subjective random variability, their distributions are computed pooling all samples of the group members. The one-sided significance of the pairwise distribution shifts (blue to red, same threshold) can be quantified using a nonparametric statistics of Brunner and Munzel [53]. This method is free of any assumption regarding the shape of the distributions. For the case with *w*(*i,j*)<0.2 the Ndim distribution of the healthy subject is significantly shifted to higher complexities compared to the distribution of the depressed subject. For the other cases (*w*(*i,j*)>0.2,0.3,0.4) the upwards shift is reversed ending at higher Ndim values for the depressed subject. For the subject-group and group-group comparisons, panels f),g), this upwards shift is weakened, we find for the *P*-values *P*_subject-subject_<*P*_subject-group_<*P*_group-group_<10^−10^. The subject-subject comparisons are in line with that of the thresholding method, but more instructive, as their statistical significance can be quantified; see [38] for applications of this statistical technique to edge distributions of weighted networks.

**Fig. 6:**
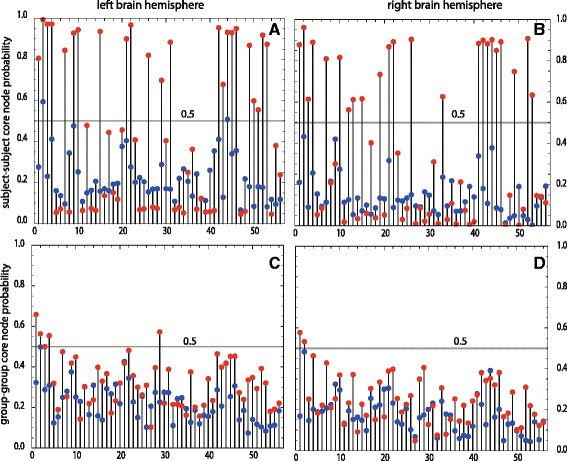
Probabilities of core nodes versus node labels. *Red* points are core nodes of depressed patients, *blue* points of healthy probands. The horizontal lines indicate probability = 0.5. Panels **a**, **b** are based on the brain hemispheres of single subjects, panels **c**, **d** are derived from groups

The calculation of Ndim is based on the maximum *k*-cliques or on the largest sets of nodes with distances ≤*k*. Maximum *k*-cliques include nodes with high functional connectivity *w*(*i,j*) or with similar resting state fMRI signals in the corresponding grey matter ROIs. Figuring out the spatial locations of maximum *k*-clique ROIs in the brain, we can deduce localized neural information. This point is complicated by the fact that several maximum *k*-cliques may exist in any binary random realization of the weighted network. For the cases with *w*(*i,j*)>0.2, we find that for depressed subjects this multiplicity (median = 11) increases compared to the multiplicity of healthy subjects (median = 6), the characteristic path length *k* (median = 2) is on the contrary rather stable. This indicates, as Ndim increases, an increasing connectivity for depression in some regions of the brain. For further analysis we focus on nodes which are contained in the intersection of all maximum *k*-cliques of a binary random realization and call these nodes core nodes. A core node is a hub node with distances ≤*k* to all maximum *k*-clique nodes of a binary network. For an ensemble of binary realizations we can quantify the probability of any node to be a core node. In Fig. 6a, b this probability is plotted for the healthy (blue) and the depressed (red) subject of Fig. 5e versus the node labels 1 to 56. A complete mapping of these labels to grey matter ROIs can be found in the Supplement of [45], the ROIs are derived from Harvard-Oxford brain atlas. This labeling is symmetric for the left and the right hemisphere of the brain. In Fig. 6c, d core node probabilities are given for the healthy group and for the group of depressed subjects of Fig. 5g. For nodes with higher probabilities, the grey matter ROIs for depressed patients are given in Table 2. Compared to the healthy subject, the probability of these nodes is strongly increased for the subject with depression (Fig. 6a, b), some of these nodes have probabilities ∼1; such nodes play in nearly all binary realizations the role of a core node or, the corresponding weighted network favors these core nodes. The clusters of core nodes with enhanced probabilities are nearly symmetric in both hemispheres indicating non-random (systematic) modifications of connectivity under depression (Figure 6a, b). For the group-group comparison, the patterns of the node probabilities (Fig. 6c, d) are similar to the subject-subject case, but the probabilities for the depressed case are weakened. The situation for the group-subject, see Fig. 5f, core nodes is not shown, but is quite similar to the subject-subject case, as can be easily inferred. Summarizing, we find that depression enhances the number of core nodes and consequently the functional connectivity between the maximum k-cliques. The grey matter core ROIs under depression which are active for all comparisons are : frontal pole (1, left, right), insular cortex (2, left, right), superior frontal gyrus (3, left), middle frontal gyrus (4, left), cingulate gyrus, anterior division (29, left), see Table 2 and Fig. 6.

**Table 2:** Core ROIs for depressed patients with probabilities as indicated

Grey matter	Subject–subject and	Group–group
ROIs (label)	group–subject (left, right)	(left, right)
	probability > 0.8	probability > 0.5
Frontal pole (1)	x (l,r)	x (l,r)
Insular cortex (2)	x (l,r)	x (l,r)
Superior frontal gyrus (3)	x (l,r)	x (l)
Middle frontal gyrus (4)	x (l,r)	x (l)
Precental gyrus (7)	x (l,r)	
Superior temporal gyrus,	x (l)	
anterior division (9)		
Superior temporal gyrus,	x (l,r)	
posterior division (10)		
Inferior temporal gyrus,	x (l,r)	
posterior division (15)		
Angular gyrus (21)	x (l,r)	
Lateral occipital cortex,	x (l,r)	
superior division (22)		
Juxtapositional lobule,	x (l,r)	
cortex (26)		
Cingulate gyrus,		x (l)
anterior division (29)		
Preconeous cortex (31)	x (l)	
Central operculum cortex (42)	x (l,r)	
Planum polare (44)	x (l,r)	
Heschl’s gyrus (45)	x (l,r)	
Planum temporale (46)	x (l,r)	
Amygdala (49)	x (l,r)	
Putamen (52)	x (l,r)	
Pallidum (53)	x (l,r)	

### Applications of functional distances

An application of functional distances is performed on correlation networks based on dual-task fMRI measurements; see Data. To keep the CPU time within limits, we reduced the number of voxels in the data mask of a subject from 60,000 to 1,208 thus eliminating coefficients in the correlation matrix. This lowers the percolation thresholds *p* and the complexity but an essential result in the work by Gallos et al. [22, 23], where separated fractal brain modules collapse for lower *p* into weakly connected non-fractal components, is still approximately valid. To compare the complexity Ndim with box-counting dimensions in such a situation, we extended the box counting algorithm for bcm to be directly applicable to the weighted network $\widetilde {W}_{n\times n}$ (path length = sum of weighted edges). Following the procedure of Gallos et al., the correlation network *W*_*n*×*n*_ of a subject is thresholded by *p* in the following way: If *w*(*i,j*)<*p* then *w*(*i,j*):=0. Then we apply the chain of transformations as described in ‘Methods’. The scaling factor *c* is calculated by

(2) $$ {\fontsize{8}{8}{\begin{aligned} {}c = \frac{k}{\max\{\widetilde{w}(i,j) < \infty : i,j = 1, \ldots n\} - \min\{\widetilde{w}(i,j) > 0 : i,j = 1, \ldots n\}} \end{aligned}}}  $$

for *k*=10^2^. In order to guarantee that $c\cdot \min \{\widetilde {w}(i,j) > 0 : i,j = 1, \ldots n\} \geq 1$ and to obtain an approximating multigraph *M*_*n*×*n*_ of $c\,\widetilde {W}_{n\times n}$, *k* must satisfy the inequality

$$k \gg \frac{\max\{\widetilde{w}(i,j) < \infty : i,j = 1, \ldots n\}}{\min\{\widetilde{w}(i,j) > 0 : i,j = 1, \ldots n\}} -1. $$

**Fig. 7:**
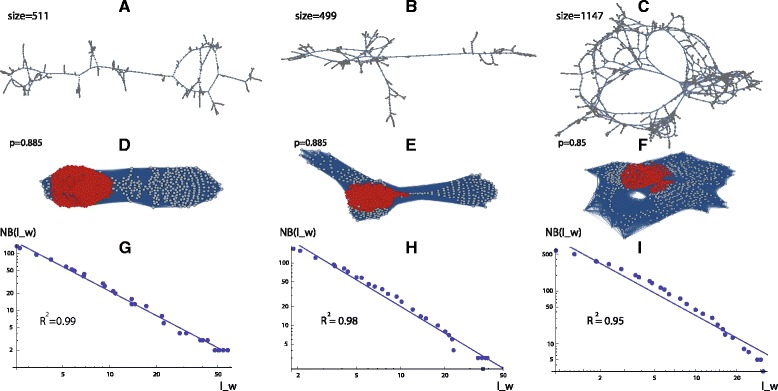
Panels **a**-**c** show the largest connected components of the $\widetilde {W}_{n\times n}$ graph (*n*=1,208) of different size according to the thresholds *p*=0.885 and *p*=0.85. Panels **d**-**f** depict the corresponding binary distance graphs *G*_*n*×*n*_ and maximum cliques. Panels **g**-**i** present the log− log plots of minimum numbers of boxes *N* *B*(*l*_*w*_) versus averaged weighted box sizes *l*_*w*_. The *R* ^2^ coefficients are also indicated. For the purposes of comparison, some estimates of the linear regressions are included

For the threshold *p*=0.885, we find two large connected components of $\widetilde {W}_{n\times n}$ of nearly equal size (size _1_=511 and size _2_=499); see Fig. 7a, b. For *p*=0.85, we find a collapsed large component in $\widetilde {W}_{n\times n}$ of size = 1147; see Fig. 7c. The corresponding binary distance graphs *G*_*n*×*n*_ including maximum cliques are shown in Fig. 7d-f. The complexities of *W*_*n*×*n*_ are Ndim_1_=1.51 and Ndim_2_=1.76, respectively. For the large component we find Ndim=2.06. The corresponding mean topological distances of $\widetilde {W}_{n\times n}$ are *μ*_1_=35, *μ*_2_=23, and *μ*=17. The log− log plots for bcm (weighted box sizes are averaged as in [33]) are shown in Fig. 7g-i. Their *R*^2^-values are *R*^2^=0.99, respectively, *R*^2^=0.98, for bcm _1_=1.43, respectively, bcm _2_≈1.41. For the collapsed component we obtain *R*^2^=0.95; the corresponding log− log plot is too non-linear to estimate a unique *FD*. These results indicate that the component of size _1_ is a fractal, the component of size _2_ may be close to a fractal, and the collapsed component is a non-fractal graph. Please note that in contrast to [22, 23], bcm is calculated by box-covering on the weighted graph $\widetilde {W}_{n\times n}$.

**Fig. 8:**
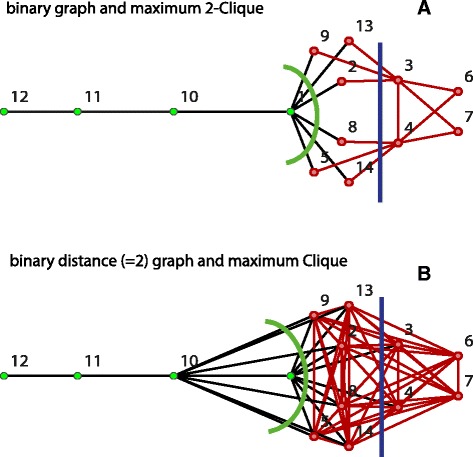
Panel **a** represents a binary graph overlaid with the maximum 2-clique (*red*). Panel **b** represents its binary distance graph for distances ≤2 and the maximum clique (*red*). Separating edge cuts are indicated by the *green* and *blue* lines

## Monotonicity and the maximum property

Monotonicity and the maximum property (see C3 and C4 in Background) are essential properties of topological or fractal dimensions [15, 54, 55]. We show that Ndim satisfies both criteria for fractal and non-fractal graphs in an appropriate way just as x-dim does for sets in $\mathbb {R}^{n}$ [13]. The detailed proofs of both theorems are given in the next section. We refer to [56] for graph-theoretic definitions and notation, and to [34] for details about weighted graphs. In the following we add parameters to Ndim in oder to clarify the interpretation.

**Monotonicity:** For induced connected subgraphs *G*_1_ and *G*_2_ of a binary or weighted connected finite graph *G*, we have the following implication.

$$G_{1} \subseteq G_{2} \quad\Rightarrow\quad {\text{Ndim}} (G_{1}, \mu, m) \leq {\text{Ndim}} (G_{2}, \mu, m), $$

where *μ* is the average and *m* is the minimum positive distance. The parameters *μ* and *m* may be derived from *G* or from *G*_*i*_. (The proofs in the next section do not depend on a specific choice of *μ* and *m*). This relation is an approximate version of Condition C3.

For infinite graphs *G*_1_ and *G*_2_ with *μ*_1_<*μ*_2_ and *μ*_1_,*μ*_2_→*∞*, the exact version of Condition 3 reads as follows:

(3) $$\begin{array}{*{20}l} G_{1} \subseteq G_{2}\quad &\Rightarrow \quad {\lim}_{\mu_{1}\to\infty} {\text{Ndim}} (G_{1},\mu_{1}, m_{1}) \\& \leq {\lim}_{\mu_{2}\to\infty} {\text{Ndim}} (G_{2},\mu_{2}, m_{2}) \end{array} $$

as

$${}{\lim}_{\mu\to\infty} {\text{Ndim}} (G_{i},\mu, m) \,=\, {\lim}_{\mu_{i}\to\infty} {\text{Ndim}} (G_{i},\mu_{i}, m_{i}), \quad i = 1,2. $$

Here it was assumed that all limits exist.

**Maximum property:** For induced connected subgraphs *G*_1_ and *G*_2_ of a binary or weighted finite connected graph *G* and for

(4) $$ {\small{\begin{aligned} {}G_{1} \uplus G_{2} := \left(V(G_{1})\cup V(G_{2}), E(G_{1})\cup E(G_{2}) \cup E(G;G_{1}, G_{2})\right), \end{aligned}}}  $$

where *E*(*G*;*G*_1_,*G*_2_) denotes the set of all edges of *G* directly connecting vertices *V* of *G*_1_ and *G*_2_, we obtain the following estimates:

$${\fontsize{8}{8}{\begin{aligned} \text{Ndim} (G_{1} \uplus G_{2}, \mu,m) - &\frac{1}{{\text{lb}} ((\lfloor\mu\rfloor + m)/m)} \\ & \leq\max\{{\text{Ndim}}(G_{1},\mu,m), {\text{Ndim}}(G_{2},\mu,m)\}\\ & \leq {\text{Ndim}} (G_{1} \uplus G_{2}, \mu,m), \end{aligned}}} $$

for fixed *μ* and *m*. Here we set lb:= log2.

The above relation for Ndim is also called the quasi maximum property in [13], implying that the complexity Ndim of a finite graph is determined approximately by a subgraph with maximum clique cardinality. The error of this approximation reduces as the average distance *μ* increases, thus approaching the exact maximum property (C4) for infinite graphs *G*_1_, *G*_2_, *G*_1_⊎*G*_2_ when *μ*_1_,*μ*_2_→*∞*:

$${\fontsize{8.4}{6}{\begin{aligned} {}{\lim}_{\mu_{12}\to\infty}& {\text{Ndim}} (G_{1} \uplus G_{2}, \mu_{12},m_{12})\\ & = \!\max\!\!\left\{{\lim}_{\mu_{1}\to\infty} {\text{Ndim}}(G_{1}, \mu_{1},m_{1}),{\lim}_{\mu_{2}\to\infty} \!{\text{Ndim}}(G_{2}, \mu_{2},m_{2})\!\right\}. \end{aligned}}} $$

The existence of all limits was assumed.

## Proofs

In this section, we proof the monotonicity and maximum properties of Ndim for binary and weighted connected finite graphs *W*. We use the transformation *W*→ multigraph *M*→ distance graph*D*→ binary distance graph *G*, as described in Application of Ndim to weighted graphs /3, and present the proofs for the maximum cliques ${\text {cl}_{\max }} (G)$. The complexity Ndim for *W* is finally estimated by

$${\text{Ndim}} (W) = \frac{\log |{\text{cl}_{\max}}(G)|}{\log\left[(\lfloor{\mu}\rfloor+m)/m\right]}, $$

where *μ* denotes the average distance and *m* the minimum positive distance derived from *D*. For graph theoretic definitions and notation, see [34, 56].

**Monotonicity:** For induced subgraphs *G*_1_ and *G*_2_ of *G*, we have the implication:

(5) $$ G_{1} \subseteq G_{2}\quad\Longrightarrow\quad |{\text{cl}_{\max}}(G_{1})| \leq |{\text{cl}_{\max}} (G_{2})|,  $$

for connected as well as disconnected subgraphs *G*_1_ and *G*_2_.

### *Proof*.

If *G*_1_=*G*_2_, the identity is true. If, w.l.o.g., *G*_1_⊂*G*_2_, then the vertex cardinality $|{\text {cl}_{\max }}(G_{1})|$ is a lower bound for $|{\text {cl}_{\max }}(G_{2})|$ implying the right-hand side of (5).

Assume now that the induced connected subgraphs *W*_*i*_⊆*W* have the property that *W*_1_⊆*W*_2_. They are transformed to induced subgraphs *G*_*i*_ in the distance graph *G* with *G*_1_⊆*G*_2_. By (5), we have Ndim(*W*_1_)≤Ndim(*W*_2_), for fixed *μ* and *m* based on *W*.

To clarify the understanding of the proof of the maximum property, a binary graph (A) with *k*=⌊*μ*⌋=2 and its binary distance graph (B) are shown in Fig. 8. The maximum 2-clique in (A) and the corresponding maximum clique in (B) have identical vertex cardinality and are indicated in red. If the weight *m*(11,10)=1 in (A) is modified to *m*(11,10)=3, we still have *k*=2 and the binary distance graph (B) becomes disconnected. Edge eliminating cuts (green and blue lines) are indicated in (A) and (B). Any cut separates a graph *G* into a pair of disjoined induced subgraphs *G*_1_ and *G*_2_ with *G*=*G*_1_⊎*G*_2_. Note that in a graph *G* several maximum cliques with identical vertex cardinalities $|{\text {cl}_{\max }} |$ may exist. Due to their distance dependence, *k*-cliques of subgraphs *G*_*i*_ may involve paths outside of *G*_*i*_, as see by the green cut in (A). If the distances in (A) are artificially restricted to *G*_*i*_, statement b) in the following Lemma may not be fulfilled: Apply, e.g., the modification *m*(11,10)=3 and the green cut. Then $(|{\text {cl}_{\max }}(G)| = 10) \!>\! \left (|{\text {cl}_{\max }}(G_{1})| = 2\right) + \left (|{\text {cl}_{\max }}(G_{2})| = 7)\right)$ in (B), where ${\text {cl}_{\max }}(G_{2}) = \{2,3,4,6,7,9,13\}$.

**Maximum property:** For induced connected subgraphs *W*_1_ and *W*_2_ of a binary or weighted finite connected graph *W* and for

$${\fontsize{8}{7}{\begin{aligned} {}W_{1} \uplus W_{2} := \left(V(W_{1})\cup V(W_{2}), E(W_{1})\cup E(W_{2}) \cup E(W;W_{1}, W_{2})\right), \end{aligned}}} $$

where *E*(*W*;*W*_1_,*W*_2_) denotes the set of all edges of *W* directly connecting vertices *V* of *W*_1_ and *W*_2_, we obtain the following estimates:

$$\begin{array}{*{20}l} {\text{Ndim}} \left(W_{1} \uplus W_{2}\right) &- \frac{1}{{\text{lb}} ((\lfloor\mu\rfloor + m)/m)} \\ &\leq\max\left\{{\text{Ndim}}(W_{1}), {\text{Ndim}}(W_{2})\right\}\\ & \leq {\text{Ndim}} (W_{1} \uplus W_{2}), \end{array} $$

for fixed *μ* and *m*. Here we set lb:= log2.

We prove the maximum property in several steps.

### **Lemma**.

Assume that *G*_1_ and *G*_2_ are induced connected subgraphs of a finite binary distance graph *G*. If *G*_1_∩*G*_2_=*∅* and *G*_1_⊎*G*_2_ is connected, then we have for the vertex-cardinalities |·| the following estimates:

- $\max \left \{|{\text {cl}_{\max }}(G_{1})|, |{\text {cl}_{\max }}(G_{2})|\right \} \leq |{\text {cl}_{\max }}(G_{1}\uplus G_{2})|$;
- $|{\text {cl}_{\max }}(G_{1} \uplus G_{2})| \leq |{\text {cl}_{\max }}(G_{1})|+ |{\text {cl}_{\max }}(G_{2})|$.

*Proof of* (a): Applying the operation ⊎ to *G*_1_ and *G*_2_ implies that *G*_*i*_⊆*G*_1_⊎*G*_2_, for *i*=1,2; see Fig. 8 for an illustration. By monotonicity, this set containment yields that $|{\text {cl}_{\max }}(G_{i})| \leq |{\text {cl}_{\max }}(G_{1}\uplus G_{2})|$, which gives statement (a).

*Proof of* (b):

- Assume that no eliminated edge connecting *G*_1_ and *G*_2_ is contained in any of the *n*≥1 maximum cliques of *G*_1_⊎*G*_2_. Then, (b) holds as an inequality; see the green cut in Fig. 8.
- Assume that only one maximum clique ${\text {cl}_{\max }}(G_{1}\uplus G_{2})$ exists and that eliminated edges connecting *G*_1_ and *G*_2_ are contained in this maximum clique ${\text {cl}_{\max }}(G_{1}\uplus G_{2})$; see blue cut in Fig. 8. Using the notation ${\text {cl}_{\max,{i}}} : = {\text {cl}_{\max }}(G_{1}\uplus G_{2})$ restricted to *G*_*i*_, we have $|{\text {cl}_{\max }}(G_{1}\uplus G_{2})| = |{\text {cl}_{\max,{1}}}$$|+|{\text {cl}_{\max,{2}}}|$ and (b) holds as an identity in case $|{\text {cl}_{\max,{i}}}| = |{\text {cl}_{\max }}(G_{i})|$, for *i*=1,2. On the other hand, (b) is satisfied as an inequality if $|{\text {cl}_{\max,{i}}}| < |{\text {cl}_{\max }} (G_{i})|$, for *i*=1 or *i*=2. If $|{\text {cl}_{\max,{i}}}|$ contains only a single vertex then $|{\text {cl}_{\max,{i}}}| = 1$.
- First assume that we have *n*>1 maximum cliques ${\text {cl}_{\max }}(G_{1}\uplus G_{2})$. For a cut that eliminates edges of *m* (*m*<*n*) maximum cliques ${\text {cl}_{\max }}(G_{1}\uplus G_{2})$, the inequality is true, as there exists at least one maximum clique ${\text {cl}_{\max }}(G_{1}\uplus G_{2})\subseteq G_{i}$ for *i*=1 or *i*=2. If the edges of all *n* maximum cliques ${\text {cl}_{\max }}(G_{1}\uplus G_{2})$ are removed, then we have for every maximum clique ${\text {cl}_{\max }}(G_{1}\uplus G_{2})$ the identity $|{\text {cl}_{\max }}(G_{1}\uplus G_{2})| = |{\text {cl}_{\max,{1}}}|+|{\text {cl}_{\max,{2}}}|$. Suppose now that there exists a maximum clique ${\text {cl}_{\max }}(G_{1}\uplus G_{2})$ with $|{\text {cl}_{\max,{1}}}| = |{\text {cl}_{\max }} (G_{1})|$. Then, $|{\text {cl}_{\max }} (G_{2})| \geq |{\text {cl}_{\max,{2}}}|$ which yields (b). On the contrary, if we assume that for all *n* maximum cliques $|{\text {cl}_{\max,{1}}}| < |{\text {cl}_{\max }} (G_{1})|$, then inequality holds in (b). $\square $

Now overlapping subgraphs are analyzed. Two cuts are involved to separate *G*_1_ and *G*_2_.

### **Remark1**.

Statements (a) and (b) in the Lemma also hold when *G*_1_∩*G*_2_≠*∅* for connected subgraphs *G*_1_ and *G*_2_.

*Proof of* (a): Follows the same arguments as those given in the proof of the lemma.

*Proof of* (b): Assume w. l. o. g. that $\widetilde {G}_{1} := (G_{1} \uplus G_{2}) - G_{2}$ is connected, where $\widetilde {G}_{1} \subset G_{1}$, $\widetilde {G}_{1}\cap G_{2} = \emptyset $, and $\widetilde {G}_{1}\uplus G_{2} = G_{1}\uplus G_{2}$. Then statement (b) in the Lemma holds. By monotonicity, (b) is then also true for the subgraphs *G*_1_ and *G*_2_. If $\widetilde {G}_{1}$ is not connected, we follow the same argumentation applying the definition of a maximum clique for disconnected binary graphs as given in Application of Ndim to weighted graphs /3c. $\square $

### **Remark2**.

Both statements in the Lemma are also valid if *G*_1_ and *G*_2_ are connected, *G*_1_∩*G*_2_=*∅*, and *G*_1_⊎*G*_2_ is disconnected.

### *Proof*.

Statements (a) and (b) hold trivially as an equality, respectively, an inequality.

### **Remark3**.

If the induced subgraphs *G*_1_ and *G*_2_ are disconnected, then statements (a) and (b) in the Lemma are valid.

### *Proof*.

Apply the argumentation given in the above proofs with trivial modifications.

### **Remark4**.

Statement (b) in the Lemma and Remarks 1, 2, or 3 imply that

$${}\mathrm{(b')}\quad|{\text{cl}_{\max}} (G_{1}\uplus G_{2})| \leq 2\,\max\{|{\text{cl}_{\max}}(G_{1})|, |{\text{cl}_{\max}}(G_{2})|\}. $$

Combining (a) and (*b*^′^) and taking the logarithm to base 2 yields the maximum property.

## Discussion

We introduced in this paper a new measure of complexity, Ndim, for binary and weighted graphs. Numerical applications to grids, fractal and non-fractal models, and to human brain data are complemented by the proofs of some general mathematical properties of Ndim. The term complexity can be understood in a picturesque manner if we regard complete graphs with finite size and constant weights *w*(*i,j*)=*c*>0. In this case, Ndim= log(size)/ log2. Such graphs combine maximum complexity with maximum cost thus indicating that for a fixed size increasing complexity may be due to a proliferation of increasing network connectivity. The concept Ndim is closely related to box-covering dimensions for fractal graphs derived from log− log plots [10, 11]. Although Ndim, if applied to fractal graphs, can be interpreted as a fractal dimension, Ndim does not rely on log− log plots, which quickly become uninformative losing linearity by hub attraction or noise.

The construction of Ndim is motivated by the extended counting method, proposed by Sandau and Kurz, see [13, 14], to calculate a fractal dimension for point sets in $\mathbb {R}^{n}$, and relies for graphs essentially on the computation of maximum *k*-cliques in binary or multi-graphs. The use of distance-based *k*-cliques to count the maximum vertex cardinality of “tightly knit vertex groups” is somewhat arbitrary; diameter-based *k*-clubs [30] might also be reasonable candidates but would require substantially longer CPU times for some of our applications. For the measure of cohesion we used *k*=⌊average distance⌋, where the average distance of a graph is a measure for its compactness. The alternative *k*=⌊diameter/2⌋, which is closer to Sandau’s original proposal was also numerically tested. We found that for graphs with small distances, Ndim computed by using *k*=⌊diameter/2⌋ lost robustness when short path graphs were added randomly.

Fractal dimensions *FD* should satisfy (at least approximately) a list of conditions given in Background. For graphs, the following conditions are relevant: *FD*s are invariant to multiplicative factors, *FD*s are extensions of topological dimensions, different *FD*s for self-similar fractal graphs are equivalent, and the monotonicity and maximum properties are satisfied (C2–C6). According to the definitions of Ndim for binary and weighted graphs, Ndim satisfies factor invariance (C2). In Ndim and manifolds, we showed that Ndim approaches the topological dimensions of grid graphs (C5) as their size increases. In addition, we quantified that Ndim converges faster than the box-covering dimension MEMB to the topological dimension of a grid. Applying cost and global efficiency to the regular latices, we find that both topological measures of complexity tend to zero as the size increases losing complexity information; see Table 1. Models of self-similar fractal binary graphs are explored with Ndim and the box-covering dimensions MEMB and bcm, in order to study the agreement between *FD*s for self-similar fractal graphs (C6). These models are iteratively generated and increase in size, diameter, and node degree, for an increasing generation parameter *g*. In the limit *g*→*∞*, a box-covering dimension of the model can be calculated analytically. We found that with increasing parameter *g* the quantities Ndim, MEMB, and bcm approach the model dimension (see Fig. 2). CPU-time limitations prevented the investigation for graphs of size >4^7^. Different degrees of hub attraction or noise destroy the fractality of a graph. The box-covering dimensions are no longer well defined in such cases in contrast to Ndim which is applicable beyond fractality. (See Fig. 3 for an exploration of non-fractal models with hub attraction).

The iteration of the graph models applies an inverted renormalization process; the growth of the parameter *g* reduces the length scale and increases the resolution of a graph. Accordingly, the iteration process can be interpreted as an increased network parcellation. We see in Fig. 3 that Ndim is for *e*>0.25 not scale invariant, especially for small-world graphs (*e*=1); however, for fractal graphs or for graphs close to fractality (*e*≤0.25) Ndim is quite stable for *g*>5, see also Fig. 2. Studying the effect of different spatial parcellations of the human brain on graph metrics is an important clinical issue [57], it relates to the problem of comparability of studies with different brain parcellations. It is shown in [57] that topological metrics of resting state fMRI networks vary with the spatial parcellation scales; however, some qualitative properties, like small-worldness or scale-freeness are stable. This differs from our results, where the metric Ndim is increasingly stable for fractal graphs. Fractal graphs were detected in the human brain by Gallos et al. [22, 23] for dual task fMRI data and convenient thresholding (see Application of functional distances); see also Fig. 7. For two different spatial brain parcellations the results were similar, see Supplement of [22]. A systematic investigation of the dependence of their findings on spatial brain parcellations was to our knowledge however not performed. Studying the dependence of complexity Ndim for real data on different brain parcellations is beyond the scope of the present paper and may be a topic of future research. At present we can only say that scale invariance of Ndim can be achieved if the spatial brain parcellation induces a network parcellation of a fractal network.

The conditions monotonicity and maximum property (C3, C4) are outlined and proven in Proofs. Monotonicity asserts that the complexity Ndim cannot decrease for graphs *G*_1_, *G*_2_ with *G*_1_⊆*G*_2_. The maximum property claims that the complexity Ndim of a graph *G* can be estimated by the complexity of a subgraph of *G*. We showed that for binary and weighted, fractal and non-fractal graphs monotonicity is approximately satisfied for finite graphs. The same conclusion holds for the maximum property. These approximations improve as the average distance increases. In the limit, as the average distance approaches infinity, conditions C3 and C4 hold exactly. Conditions C3, C4 are essential properties of any concept of dimension [15, 54, 55], may it be topological or fractal. As Ndim satisfies conditions C2-C6 at least approximately, we may interpret Ndim when applied to fractal graphs as a *FD*, similar to MEMB or bcm. More generally, without restricting to fractal graphs, we may interpret Ndim as a dimension measuring complexity.

Bio-medical networks are frequently modeled by weighted graphs. Methods to apply Ndim to weighted graphs were presented in Application of Ndim to weighted graphs. All three methods mapped the weighted graph to convenient binary graphs, where a maximum *k*-clique was computed. Thresholding proved to be the fastest method, but simplifies any weight *w*(*i,j*)>0 to a yes-no edge *a*(*i,j*)=1 or 0. Comparing with cost and efficiency, we find that Ndim has enhanced power to differentiate connectivity in a healthy and a depressed subject; see Fig. 4. More information about the weights is maintained in the Monte Carlo method, where *w*(*i,j*) is mapped to an ensemble of binary edges; the higher *w*(*i,j*) the more frequently *a*(*i,j*)=1 is sampled. Mapping a weighted graph to an ensemble of binary random graphs implies that the complexity of the weighted graph is described by a distribution of complexities Ndim of the corresponding binary graphs; see Fig. 5. Therefore, the difference of the complexity between two weighted graphs can be made statistically relevant by significance testing, analyzing the shift in the two Ndim distributions [53]. This may be of importance for a personalized clinical analysis of the abnormal functional connectivity under depression. As Ndim is a regional measure, core nodes within the maximum *k*-cliques can be detected to localize ROIs which act as communication centers or hubs. Our analysis shows that the probability for such hubs is increased for subjects under depression; see Fig. 6.

In a third approach, using functional distances, the weighted graph was transformed to a binary *k*-distance graph and Ndim was calculated via the cardinality of its maximum 1-clique. We applied this method to a dual-task fMRI data set [22, 23] and compared Ndim with the box-covering dimension bcm that was modified for a direct application to weighted graphs. For a high percolation threshold (*p*=0.885) we found, similar to Gallos et al. [22, 23], large connected components which are at least close to a fractal with rather low complexities Ndim and bcm. For a lower threshold (*p*=0.85), the components collapse into a non-fractal network with lower average distance and higher complexity Ndim. Due to the non-linear log− log plot for *p*=0.85, bcm can no longer be calculated reliably over the entire range of box sizes. This demonstrates the advantage of Ndim, which can quantify complexity beyond fractality. The limited applicability of the box-covering dimension bcm is apparent already for *R*^2^ coefficients with *R*^2^<0.98; see Fig. 7.

The weights of a network can be altered by the application of thresholding techniques or by noise. Direct thresholding is applied in Figs. 4, 5 and 7. In all cases correlation thresholding modifies the network complexity, in Fig. 7 even the type – it separates strongly connected fractal subgraphs from a mixed compound. This influence of the threshold on complexity is intuitively clear: Different correlation thresholds in, e.g., fMRI data, focus on signals with a different degree of similarity; the lower the threshold, the higher the complexity due to monotonicity (See Fig. 4). In some studies correlation thresholding is additionally constrained by the condition of identical cost for the pair to be compared. We reparametrized the results of Fig. 4 and achieved for cost =0.1,0.2,0.3,0.4 for efficiency and Ndim smaller differences between healthy and depressed patients compared to direct correlation thresholding; all differences of efficiency were <0.01, the differences of Ndim were 0.08,0.1,0.5, 0.58, respectively. We still find that Ndim has more informative power than efficiency. Next we look at robustness or noise sensitivity of Ndim. To test this, the weights of the correlation graph for Fig. 4 (threshold >0.2) were modified by adding uniformly distributed noise. For *w*(*i,j*)→*w*(*i,j*)+*ε*(*i,j*), *ε*(*i,j*)∈[−*w*(*i,j*)·*α*,*w*(*i,j*)·*α*], where *α*∈{0.1,0.2,0.3,0.5}, we obtained Ndim=3.7→Ndim= 3.7, 3.6, 3.6, and 3.3, respectively. Under the influence of such noise, Ndim is quite robust. Different is the effect of noise on binary graphs by adding randomly connections =1; e.g. complexity Ndim of the fractal model *e*=0, *g*=6 of Fig. 3 increases by 10,20,30 *%* if only 1,2,3 *%* random connections are added, see also [12].

Computational cost (CPU time) for large networks is a problem for any NP complete algorithm [30]. In our calculations of Ndim for the synthetic networks of Fig. 3 CPU times depend on the type characterized by parameter *e* and on the generation parameter *g* or size. We find for any type monotonicity of Ndim with size. This agrees with intuitive expectation, as renormalization (transition *g*→*g*−1) induces blurring reducing vertices and low distance connectivities; if renormalization produces a subgraph (*g*−1) of the graph (*g*) monotonicity of Ndim could be proven formally. The worst case (*g*=7, size =16.384) CPU times for the graphs with *e*=1,0.75,0.5 are CPU = 86, 69, 590 sec, for *g*=5 (size =1.024) CPU ∼1 sec in all cases. Critical are the cases *e*=0.25 and 0. We find, e.g., for the case *e*=0.25 and *g*=7 a CPU time of more than 6 hours, for *g*=4,5,6, a CPU time of only 1,1,10 sec. The mentioned CPU times are calculated without any constraints on the clique cardinalities; especially constraining the lower bound can improve speed [31]. Monotonicity enables a simple rule to estimate this lower bound exp(Ndim(*g*−1)· log(*k*(*g*)+1))=lower bound of| max*k*-clique(*g*)|. If we incorporate this information as a constraint in our calculations we find the following CPU times: for *e*=0.25 and *g*=7 CPU =1650 sec, for *e*=0 (fractals) and *g*=5,6,7 CPU =1,30,1560 sec. For fractal networks based on real data, as used in Fig. 7a,b, we needed only CPU ∼2 sec without constraint. This may be due to inherent type mixing or noise in real data networks. We tested computational cost for seven additional connectivity networks taken from the brain-connectivity-toolbox, see Data. We had to symmetrize some of the adjacency matrices, as Ndim is defined for undirected networks only, using the triangle above the diagonal of the matrix. In some cases the graphs were disconnected, in such cases the largest connected component was used. For these 7 networks complexity was calculated without constraints, CPU time was in all cases moderate, see Table 3. All CPU times were produced by a single processor (2.8 GHz) applied to the commercial software Mathematica 9 (constraining is implemented) and should be regarded only as rough estimate which may be improved by faster hard ware equipment or software. A more basic improvement is possible by application of parallel computing as was demonstrated in [58], where fast algorithms for the calculation of the maximum clique were developed and tested; we showed in Application of Ndim to weighted graphs that Ndim can always be calculated by a maximum clique (*k*=1). Applying 128 processors to the maximum clique calculation for a network a speeding up factor of ∼20 could be achieved. Implementation of this involved method was beyond our scope.

**Table 3:** Binary networks from the brain-connectivity-toolbox

Name	Size	CPU (sec)	Ndim
Autobahn	1158	377	2.12
Air500	500	6	4.95
Celegans277	261	<1	3.37
Celegans131	125	<1	2.84
Mac95	92	<1	4.4
Macaque71	71	<1	3.06
Macaque47	47	<1	2.81

## Conclusion

We presented a new measure, Ndim, to quantify complexity originating by the proliferation of edge-connectivity in binary or weighted networks. Ndim is essentially determined by the cardinality of a maximum *k*-clique of the graph and fulfills the conditions of a dimension. These dimensional properties guarantee a large informative power of Ndim, compared to cost and efficiency. In addition, for a fractal graph Ndim estimates its fractal dimension, like the recently proposed box-covering dimensions. But, box-covering dimensions cannot be calculated uniquely for fractals perturbed by noise caused by the addition of random edges or for graphs with hub attraction like small world graphs. For Ndim however, there is no such limitation; comparisons of complexity between all types of finite graphs can be performed. These features were demonstrated for model calculations and by comparisons of functional brain connectivity for healthy and depressed subjects. Due to this informative power and flexibility, Ndim may become a useful tool in biomedical studies performing comparisons of complexity of finite networks.
